# Aerobic Exercise and Pharmacological Treatments Counteract Cachexia by Modulating Autophagy in Colon Cancer

**DOI:** 10.1038/srep26991

**Published:** 2016-05-31

**Authors:** Eva Pigna, Emanuele Berardi, Paola Aulino, Emanuele Rizzuto, Sandra Zampieri, Ugo Carraro, Helmut Kern, Stefano Merigliano, Mario Gruppo, Mathias Mericskay, Zhenlin Li, Marco Rocchi, Rosario Barone, Filippo Macaluso, Valentina Di Felice, Sergio Adamo, Dario Coletti, Viviana Moresi

**Affiliations:** 1 DAHFMO Unit of Histology and Medical Embryology, Interuniversity Institute of Myology, Sapienza University of Rome, Italy; 2 Department of Kinesiology, Research Group in Exercise Physiology, KU Leuven, Belgium; 3 Department of Mechanical & Aerospace Engineering, Sapienza University of Rome, Italy; 4 Department of Biomedical Sciences, University of Padua, Italy; 5 Ludwig Boltzmann Institute of Electrical Stimulation and Physical Rehabilitation, Department of Physical Medicine and Rehabilitation, Wilhelminenspital Wien, Austria; 6 IRCCS Fondazione Ospedale San Camillo, Venezia, Italy; 7 Department of Surgical and Gastroenterological Sciences, University of Padua, Italy; 8 Department of Biological Adaptation and Ageing B2A (CNRS UMR 8256 - INSERM ERL U1164 - UPMC P6), Pierre et Marie Curie University, Paris 6, France; 9 Department of Biomolecular Sciences, Unit of Biostatistics, “Carlo Bo” University of Urbino, Italy; 10 Department of Experimental Biomedicine and Clinical Neurosciences (BioNeC), University of Palermo, Italy

## Abstract

Recent studies have correlated physical activity with a better prognosis in cachectic patients, although the underlying mechanisms are not yet understood. In order to identify the pathways involved in the physical activity-mediated rescue of skeletal muscle mass and function, we investigated the effects of voluntary exercise on cachexia in colon carcinoma (C26)-bearing mice. Voluntary exercise prevented loss of muscle mass and function, ultimately increasing survival of C26-bearing mice. We found that the autophagic flux is overloaded in skeletal muscle of both colon carcinoma murine models and patients, but not in running C26-bearing mice, thus suggesting that exercise may release the autophagic flux and ultimately rescue muscle homeostasis. Treatment of C26-bearing mice with either AICAR or rapamycin, two drugs that trigger the autophagic flux, also rescued muscle mass and prevented atrogene induction. Similar effects were reproduced on myotubes *in vitro*, which displayed atrophy following exposure to C26-conditioned medium, a phenomenon that was rescued by AICAR or rapamycin treatment and relies on autophagosome-lysosome fusion (inhibited by chloroquine). Since AICAR, rapamycin and exercise equally affect the autophagic system and counteract cachexia, we believe autophagy-triggering drugs may be exploited to treat cachexia in conditions in which exercise cannot be prescribed.

Cancer is a major, worldwide health problem that is responsible for one in four deaths^1^,^2^. Numerous studies have investigated the association between physical activity and a lower risk of developing cancer. A striking finding that recently emerged from both epidemiological studies and clinical trials is that the improved prognosis observed in physically active cancer patients is due to the exercise performed after cancer diagnosis as opposed to exercise habits before the disease^3^,^4^. Depending on the tumor type, 9 to 18 metabolic equivalent task (MET) hours per week of physical activity yielded significant benefits when compared with a sedentary lifestyle (9 MET hours/week are equivalent to 5 weekly 30-minute sessions of brisk walking)^5^. While physical activity following diagnosis lowers the risk of both cancer-specific and overall mortality, physical activity spontaneously declines in cancer patients and such patients do not exercise enough even after tumor resection^6^. Although exercise increases survival in cancer patients and improves their quality of life^7^, prescribing exercise to these patients is not a straightforward task. It is, thus, of paramount importance to identify potential pharmacological treatments that may be able to mimic the effects of exercise in pathological conditions. While clinical studies have shown that exercise has a highly beneficial effect on cancer patients, it should be borne in mind that such studies are observational by nature, and that the cause and effect cannot consequently be assumed; in addition, they fail to provide insights into possible mechanisms that could be exploited for pharmacological treatments.

A major cause of patient mortality, morbidity and low quality of life, including exercise intolerance, is cancer-associated cachexia. Cachexia is defined as “a complex metabolic syndrome associated with underlying illness and characterized by loss of muscle with or without loss of fat mass. Anorexia, inflammation, insulin resistance and increased muscle protein breakdown are frequently associated with wasting disease”^8^,^9^. Cachexia is not a condition that is exclusive to cancer, it being shared by several chronic diseases and severe acute injuries. Cachexia bears some similarities to sarcopenia and other forms of muscle atrophy, even though it remains distinct at both the morphological and molecular levels^10^. Cancer cachexia is probably the most severe form of muscle atrophy and is associated with increased morbidity and mortality^10^. In the case of treated cancers, chemotherapy per se induces muscle wasting regardless of tumor progression, thereby exacerbating cachexia and worsening the patient’s quality of life; cachexia, in turn, interferes with the efficacy and practicability of chemotherapies. We and others have demonstrated that the expression of both muscle fiber and satellite cell genes are affected in cachexia, clearly indicating that cancer-induced muscle wasting does not result from a general loss of muscle proteins but derives from the selective dysregulation of key muscle gene products^11^. Several therapeutic strategies have been adopted in an attempt to counteract cancer cachexia, to improve the quality of life and to increase life expectancy of patients^12^. However, therapeutic approaches based on a single target, such as the use of recombinant antibodies against tumor necrosis factor, have failed so far, the most likely cause being that cancer cachexia is triggered by multiple factors.

Exercise training has long been proposed to counteract cachexia. Indeed, stimulation of muscle contraction has been shown to reduce muscle wasting in tumor-bearing animals^13^. The molecular bases of skeletal muscle adaptation to exercise have been extensively reviewed^14^. In tumor-bearing mice, muscular damage may arise from tumor-induced dysregulation of dystrophin expression^15^, which increases muscle fragility. Since exposing tumor-bearing mice, and possibly even humans, to exercise protocols might be deleterious, pharmacological alternatives to physical activity are needed.

In this regard, pharmacological treatments are being proposed to mimic the beneficial effects of exercise in an attempt to replace or supplement exercise-training programs. These compounds, which are referred to as “exercise mimetics”^16^,^17^, may represent a very promising tool for the treatment of severe muscular and non-muscular pathologies^16^,^18^. Indeed, they induce muscle responses that are similar to those mediated by endurance exercise^17^. In particular, the AMP analog 5-aminoimidazole-4-carboxamide-1-beta-D-ribofuranoside (AICAR) is an AMP-activated protein kinase (AMPK) activator that increases endurance in sedentary mice by reprogramming muscle metabolism^17^. AICAR mimics the typical increase in the AMP/ATP ratio that occurs following exercise, ultimately activating the AMPK; hence, the expression “exercise mimetics”^16^. Indeed, in response to exercise or exercise mimetics, AMPK integrates multiple transcriptional programs by interacting with transcriptional regulators of the metabolism, such as peroxisome proliferator-activated receptor gamma coactivator 1a (PGC1a) and peroxisome proliferator-activated receptor (PPAR), and by inhibiting the mammalian target of rapamycin (mTOR) signaling^18^. Moreover, by opposing mTOR signaling^19^ and by activating FoxO3a^20^, AMPK promotes skeletal muscle autophagy. The inhibition of mTOR mediates many of the effects of rapamycin, another drug used as an immunosuppressant and disruptor of signaling controlling muscle homeostasis^21^. Rapamycin also stimulates autophagy^22^.

Autophagy takes place at basal levels in all eukaryotic cells, turning over long-lived macromolecules and large supramolecular structures. The excessive activation of autophagy aggravates muscle wasting^23^,^24^. By contrast, inhibition of lysosome-dependent degradation causes myopathies such as Pompe and Danon diseases, and autophagy inhibition is believed to play a role in many myopathies characterized by inclusion bodies or abnormal mitochondria, as well as in dystrophies^25^,^26^. Accordingly, autophagy is required to maintain muscle mass^27^.

Here we present data showing that cancer triggers the induction of the autophagic markers LC3b and p62/SQSTM 1 in skeletal muscle in both mice and carcinoma patients. This scenario is deleterious to muscle as well as to other tissues^28^. Aerobic exercise and pharmacological treatments that promote autophagy restore muscle homeostasis and modulate autophagy in cancer cachexia. These observations shed light on a possible mechanism underlying the beneficial effects observed in cancer patients who perform a moderate amount of physical activity, and highlight the role of autophagy-triggering drugs as a potential therapeutic approach to treating cancer patients.

## Results

### Voluntary wheel running counteracts cancer cachexia

We allowed healthy and C26-bearing mice to perform voluntary wheel running (WR) by equipping their cages with a wheel, to avoid muscle damage resulting from imposed, forced exercise protocols such as a treadmill. None of the tumor-bearing mice interrupted the running activity as the disease progressed, which suggests that they were not subjected to major discomfort. Nonetheless, we measured muscle damage in both control and C26-bearing mice, which underwent or did not undergo two different routines of voluntary physical activity (5 and 19 days of WR), to determine whether WR exacerbates muscle damage in cachexia. By quantifying Evans’ Blue dye (EBD) uptake by two different muscles, which is considered a marker of cell impairment, we confirmed that the C26 tumor induces muscle fiber damage, and we found that WR per se did not damage or interact with tumor-induced muscle fiber damage (Supplementary Fig. S1). Running activity was monitored daily by measuring several parameters, including the running distance, in both control and C26-bearing mice (Table 1). The results show that C26-bearing mice were still capable of running, though to a lesser extent than control mice but at a similar intensity.

To quantify the effects of WR on parameters commonly used as diagnostic criteria for cachexia^8^, we compared several groups of animals: control (i.e. healthy) mice in the absence and presence of WR, and C26 tumor-bearing mice in the absence and presence of WR and of pharmacological treatments used as replacements for WR. In terms of final weight (i.e. carcass weight, which is the body weight minus tumor weight), we found no effect of WR on healthy mouse body weight; instead, all the treatments (i.e. WR and drug administrations) affected body weight by interfering with the C26-induced body weight loss (Table 2).

**Figure 1:**
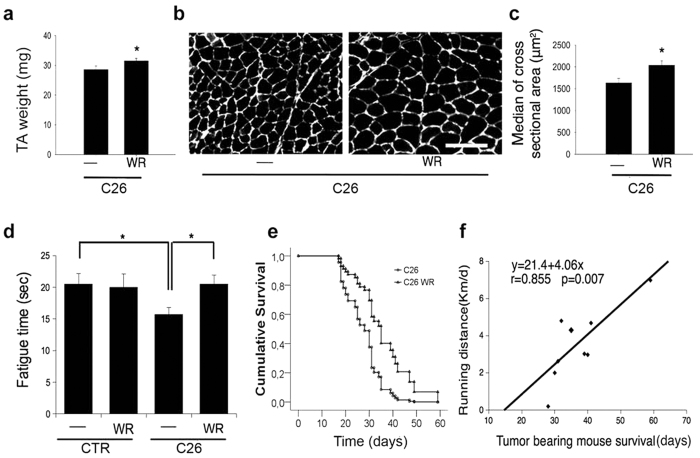
Voluntary wheel running counteracts cancer cachexia and prolongs survival of tumor-bearing mice. (**a**) TA muscle weight of C26-bearing mice in the absence (—) or presence (WR) of voluntary wheel running for 19 days. WR rescues muscle mass of C26-bearing mice. Data are shown as mean ± SEM; 6 < n < 9 for each group, *p < 0.05 by Student’s t-test. (**b**) Representative immunostaining for laminin on TA muscle of C26-bearing mice in the absence (—) or presence (WR) of wheel running for 19 days, showing that WR increases fiber size and improves basement membrane morphology in C26-bearing mice. Scale bar = 100 microns. (**c**) Median ± SEM of glycolytic TA myofiber cross-sectional area of C26-bearing mice in the absence (—) or presence (WR) of wheel running for 19 days. WR rescues fiber size of C26-bearing mice. n = 5 for each group, *p < 0.05 by Student’s t-test. (**d**) Fatigue time of EDL muscle of control or C26-bearing mice in the absence (—) or presence (WR) of wheel running for 19 days. Data are shown as mean ± SEM. Two-way ANOVA (F = 5.77; df 1; p = 0.025) shows an interaction between the negative effect of C26 tumor and wheel running, which restores fatigue time to control levels; data are shown as mean ± SEM; 6 < n < 9 for each group, *p < 0.05 by Tukey’s HSD test. (**e**) Survival curves derived from Cox model for statistical analysis of C26-bearing mice in the absence (C26, circles) or presence (C26 WR, triangles) of wheel running. Wheel running significantly (p < 0.041) increased survival of C26-bearing mice. n = 9 for each group. (**f**) Linear correlation between running distance (Km per day) and life span (days of survival) in C26-bearing mice. From the curve equation, one can infer that each km/day of wheel running corresponds to an increase of approximately 4 days in life span.

Since WR prevented the severe body weight loss that characterizes cachexia and since skeletal muscle mass accounts for about 50% of the body mass, a rescue of muscle homeostasis was a predicted outcome. Thus, we measured the effects of exercise on a specific representative muscle, i.e. the tibialis anterior (TA), and found that WR increased TA mass in C26-bearing mice (Fig. 1a). Histological and morphometric analyses of the muscle fiber cross-sectional area (CSA), performed by using immunostaining for laminin to visualize the myofiber perimeter, revealed a significantly bigger CSA in exercised C26-bearing mice than in sedentary C26-bearing mice (Fig. 1b,c).

Along with muscle mass, WR improved muscle function in C26-bearing mice: the extensor digitorum longus (EDL) muscle, a muscle that we previously found to be affected in cancer cachexia^29^, exhibited a shorter fatigue time (defined as the time required to halve the value of the muscle maximum strength) in C26-bearing control mice than in non-C26-bearing control mice, while no differences were detected in WR C26-bearing mice (Fig. 1d). A negative interaction between the C26-induced decrease in fatigue time and WR was found, which thus indicates that WR rescues muscle function in C26-bearing mice.

Given the many positive effects exerted by WR on skeletal muscle homeostasis and function, we sought to verify whether WR might extend the life span of C26-bearing mice. Since the presence of a wheel in the cage may represent a form of environmental enrichment^30^, we also included, as an additional control, treatments designed to test whether environmental enrichment had significant effects per se on mouse survival. Thus, we combined the use of regular or environmentally-enriched (EE) cages (Supplementary Fig. S2a) with the absence or presence of the wheel to evaluate the survival of C26-bearing mice. WR significantly increased survival in C26-bearing mice whereas EE cages alone did not (Supplementary Fig. S2b). We also observed that the effects of WR were maintained in EE cages (not shown in the figure), thus suggesting that WR and environmental enrichment do not affect each other.

WR activity increased the life span of C26-bearing mice by about 44%, thus indicating that voluntary physical activity had a marked beneficial effect on animal models of cancer cachexia, as previously reported in clinical studies. In order to assess any correlation between individual variations in the amount of exercise with output in terms of survival, we measured the daily distance covered by WR and the lifespan of the corresponding mouse in a subset of randomly chosen animals. This approach revealed a striking, linear correlation between km/day run on the wheel and survival time: each km/day of running activity was found to correspond to four additional days of survival in C26-bearing mice (Fig. 1f).

In keeping with all the aforementioned findings, WR was also found to be associated with a significant increase in food intake in WR C26-bearing mice compared with control C26-bearing mice (Supplementary Fig. S3). Moreover, the fact that food intake in C26-bearing mice did not change over time indicates that this tumor does not induce anorexia in mice as it progresses.

### Voluntary wheel running counteracts alterations in catabolic pathways in colon carcinoma (C26)-bearing mice

**Figure 2:**
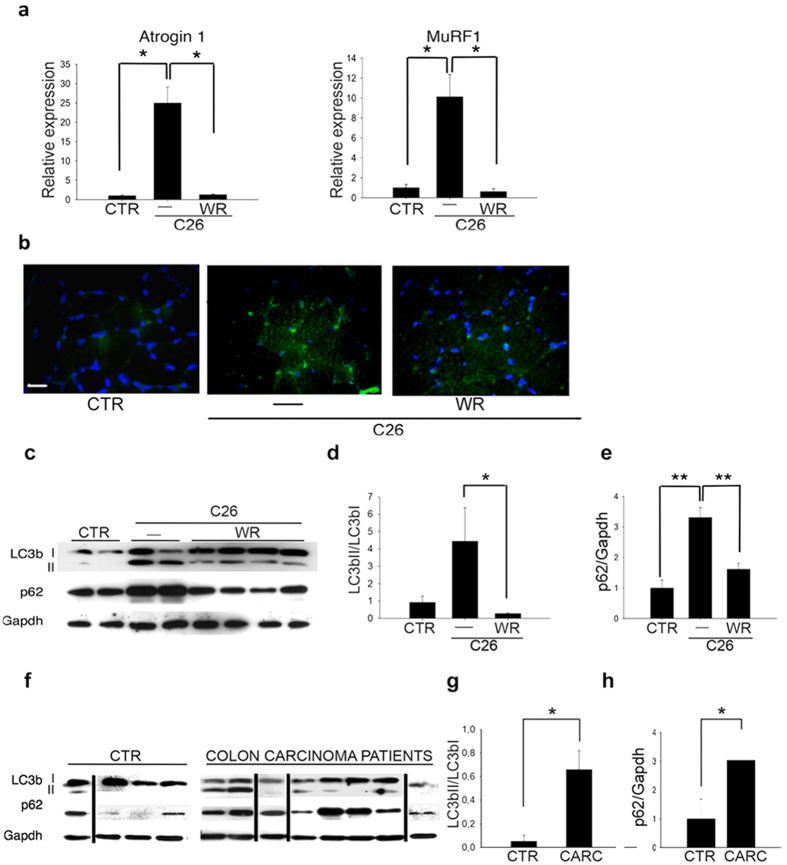
Wheel running counteracts tumor-induced atrogene expression and restores the autophagic marker levels, which are altered in both murine and human skeletal muscle in cancer cachexia. (**a**) Expression of Atrogin 1 and MuRF1 genes by real-time RT-PCR, in mice in the absence (—) or presence (WR) of wheel running, 19 days after transplantation in C26-bearing mice and in control mice (CTR). Values were normalized to Gapdh expression. Data are presented as mean ± SEM. Kruskal-Wallis test revealed a global significant difference between the three groups, followed by post-hoc test *p < 0.05. (**b**) Representative immunostaining for p62 on TA muscle of C26-bearing mice in the absence (—) or presence (WR) of wheel running for 19 days, showing an increase of p62 induction in C26-bearing mice. Scale bar = 20 microns. (**c**) Western blot analysis of LC3bI, LC3bII and p62 expression in control mice (CTR), or mice in the absence (—) or presence (WR) of wheel running, 19 days after C26 transplantation. Gapdh was used as a loading control. Densitometric analyses of Western blot showing LC3bII/LC3bI ratio (**d**) and p62/Gapdh (**e**) ratio in mice in the absence (—) or presence (WR) of wheel running, 19 days after C26 transplantation, compared with control mice (CTR). Data are presented as mean ± SEM. F = 4.63; df 2; p < 0.05 and F = 14.18; df 2; p < 0.02 by ANOVA; *p < 0.005 and **p < 0.005 by Tukey’s HSD test. (**f**) Expression of LC3b and p62 by Western blot analyses in skeletal muscle biopsies from colon carcinoma patients and healthy (CTR) subjects. Gapdh was used as a loading control. The lanes were run on the same type of gel but were non-contiguous, as indicated by the thin black lines. Densitometric analyses of Western blot showing significantly higher expression of LC3bII/LC3BI ratio (**g**) and p62/Gapdh ratio (**h**) in skeletal muscle biopsies from colon carcinoma patients (CARC), compared with healthy subjects (CTR). Data are presented as mean ± SEM. *p < 0.05 by Student’s t-test.

Cachexia is characterized by an up-regulation of the muscle specific E3-ubiquitin ligases, atrogin 1 and MuRF1^31^. When we quantified the effects of both C26 and WR on these markers in skeletal muscle, we found that both ubiquitin ligases were significantly up-regulated in muscle from C26-bearing mice when compared with control healthy mice, and that voluntary wheel running prevented the induction of these genes and consequently restored expression levels to those of controls (Fig. 2a).

It has recently been suggested that autophagy contributes to the pathogenesis of skeletal muscle wasting in cancer cachexia^32^. Using electron microscopy, we detected autophagosome-like structures in skeletal muscle fibers of C26-bearing mice (Supplemental Fig. S4), which is consistent with similar morphological findings observed at the ultrastructural level in other muscle pathologies^33^.

As aerobic exercise is known to trigger autophagy in skeletal muscle^34^, we decided to evaluate autophagy in our experimental conditions by immunostaining for p62 and Western blot analyses for LC3b and p62, as markers of autophagic induction. Immunofluorescence analyses on TA muscles detected accumulation of p62 in C26-bearing mice, if compared with control or WR C26-bearing mice (Fig. 2b). The activation of autophagy drives the processing of LC3bI into its lipidated, autophagosome-bound form LC3bII, with a high LC3bII/LC3bI ratio being considered to indicate an increased number of autophagosomes. We observed an increase in the levels of the LC3bII/LC3bI and p62/Gapdh ratio in C26-bearing mice (Fig. 2c–e), compared with control healthy mice, which is in agreement with findings by Penna *et al*.^32^. Interestingly, WR significantly reduced the LC3bII/LC3bI and p62/Gapdh ratio in C26-bearing mice (Fig. 2d,e). These findings confirm that autophagy is activated in sedentary C26-bearing mice; nevertheless, the sustained expression of p62 suggests that the autophagic flux may, despite not being blocked completely, proceed at a slower rate. Voluntary WR exercise appears to restore basal levels of autophagy, which is beneficial to muscle homeostasis insofar as it is associated with the recovery of muscle mass and function.

To validate previous observations yielded by the C26-bearing mouse model, we compared the levels of expression of the aforementioned autophagy-related proteins with data obtained from skeletal muscle biopsies of colon carcinoma patients. Remarkably, accumulation of the lipidated form of LC3b, a high LC3bII/LCb3I ratio and significantly higher p62 levels were detected in skeletal muscle of colon carcinoma patients compared with healthy subjects (Fig. 2f–h). The pathological classification of tumors, gender, age, BMI were recorded for this cohort of patients, together with the levels of the following parameters: total proteins, albumin and transthyretin, creatine kinase, C-reactive protein and leukocytes (Supplementary Tables 1 and 2). Other events and chemotherapy treatments were also taken into account. No significant correlation between p62 levels and any of these parameters was found. The lack of significant differences between patients suggests that it is possible to pool patients for the p62 analysis.

By showing that both tumor-bearing mice and patients display altered autophagic markers, our results indicate that such alterations in the autophagic system may play a role in the development of cachexia. This prompted us to further investigate the efficacy of pharmacological treatments designed to trigger autophagy in cancer-induced cachexia.

### Drugs promoting autophagy counteract body weight loss in cancer cachexia

**Figure 3:**
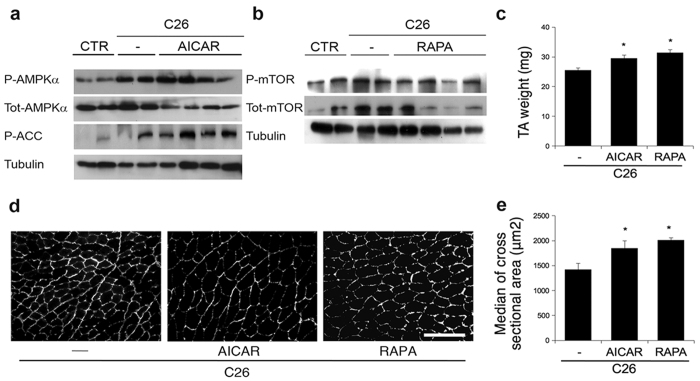
AICAR or rapamycin treatment counteracts muscle wasting in cancer cachexia. (**a**) Western blot showing total and phosphorylated AMPK levels, as well as ACC activation (p-ACC) in AICAR-treated mice, and (**b**) total and phosphorylated mTOR levels in rapamycin-treated mice (RAPA), compared with vehicle-treated mice (—) as well as untreated mice (CTR). Treatments consisted in daily IP injection of vehicle or [250 mg/Kg] AICAR (AICAR) or [2 mg/Kg] rapamycin (RAPA) for 19 days. (**c**) TA muscle weight of C26-bearing mice in the absence (—) or presence of treatment as indicated. ANOVA (F = 9.47; df 2; p < 0.0005) revealed a significant effect of the treatments on TA weight. (**d**) Representative immunostaining for laminin on TA muscle of C26-bearing mice in the presence of the treatments indicated, showing increased fiber size in treated animals. Scale bar = 100 microns. (**e**) Median ± SEM of TA myofiber cross-sectional area of C26-bearing mice in the presence of the treatments indicated. ANOVA (F = 6.86; df 2; p < 0.008) revealed a significant effect of treatments on muscle fiber CSA (F, see results). Data are shown as mean ± SEM; 12 < n < 26 for each group, * p < 0.05 by Tukey’s HSD test.

As described above, AICAR is a pharmacological activator of AMPK^17^ that has been shown to recapitulate the molecular response of skeletal muscle to aerobic exercise and to promote autophagy^14^,^35^. We therefore treated, on a daily basis, C26-bearing mice with AICAR, or a vehicle for controls, by means of intraperitoneal injections. We confirmed the activation of AMPK and its downstream target ACC in skeletal muscles of AICAR-treated mice by Western blot analyses (Fig. 3a). Rapamycin, a specific inhibitor of mTOR, is another drug known to trigger autophagy in many cell types, including skeletal muscle^36^. In parallel experiments, we treated, on a daily basis, C26-bearing mice with rapamycin, or a vehicle for controls, by means of intraperitoneal injections, and confirmed the inhibition of mTOR in skeletal muscles of rapamycin-treated mice, by Western blot analyses (Fig. 3b). Treatment with AICAR prevented weight loss in C26-bearing mice, compared with vehicle-treated C26-bearing mice (Table 2). Accordingly, AICAR blocked skeletal muscle wasting in C26-bearing mice by restoring both muscle mass and myofiber size (Fig. 3c–e). Similarly, rapamycin treatment significantly affected cancer cachexia in C26-bearing mice by preventing a drop in body weight (Table 2), which in turn resulted in heavier and larger TA muscles than in vehicle-treated mice (Fig. 3c–e). These drugs were found to exert a significant effect on both the TA weight and muscle fiber CSA, thereby showing that pharmacological treatments are highly effective in counteracting muscle wasting in C26-bearing mice. Interestingly, no difference was detected between AICAR and rapamycin treatment in terms of body weight rescue in tumor-bearing mice (Table 2).

### AICAR and rapamycin counteract muscle atrophy and affect autophagy

**Figure 4:**
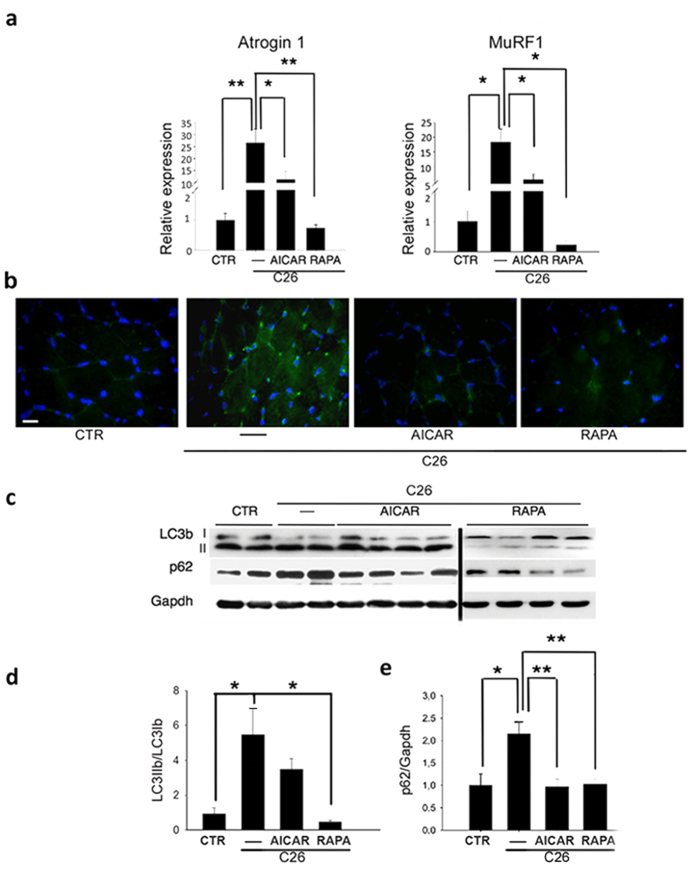
AICAR or rapamycin treatment counteracts the induction of atrogene expression and modulates autophagic markers in cancer cachexia. (**a**) Expression of Atrogin 1 and MuRF1 genes as shown by real-time RT-PCR, in mice that received daily IP treatment with vehicle (—), AICAR (AICAR) or rapamycin (RAPA), for 19 days after C26 implant, compared with control mice (CTR). Values were normalized to Gapdh. ANOVA (F = 10.08; df 3; p < 0.01;F = 7.90; df 3; p < 0.01; for Atrogin 1 and MuRF1, respectively) showed a significant effect of the treatments on ubiquitine-ligase relative expression. (**b**) Representative immunostaining for p62 on TA muscle of C26-bearing mice in the absence (—) or presence of the treatments indicated, showing an increase in p62 induction in C26-bearing mice. Scale bar = 20 microns. (**c**) Western blot analysis of LC3bI, LC3bII, p62 and Gapdh expression in the presence of the treatments indicated. The lanes separated by the thin black lines were run at the same time on parallel gels and were, therefore, non-contiguous. (**d**) Densitometric analyses of Western blot showing LC3bII/LC3bI ratio in mice in the presence of the treatments indicated, compared with control mice (CTR). ANOVA (F = 6.26; df 3; p < 0.01) revealed a significant effect of the treatments on the LC3bII/LC3bI ratio. (**e**) Densitometric analyses of Western blot showing p62 expression in mice in the presence of the treatments indicated, compared with control mice (CTR). Values were normalized to Gapdh. ANOVA (F = 5.92; df 3; p < 0.008) revealed a significant effect of treatments on p62 protein expression. Data are presented as mean ± SEM. *p < 0.05; **p < 0.005 by Tukey’s HSD test.

We compared the transcriptional response to cancer cachexia in C26-bearing mice treated with either AICAR or rapamycin with that in vehicle-treated mice 19 days after tumor transplantation. Strikingly, both AICAR and rapamycin treatment significantly reduced atrogin 1 and MuRF1 expression levels, which were instead up-regulated in vehicle-treated C26-bearing mice (Fig. 4a). Similarly to voluntary wheel running, we found that both AICAR and rapamycin treatments modulated autophagy in C26-bearing mice by significantly reducing C26-mediated p62 accumulation, as revealed by immunofluorescence analyses on TA muscles (Fig. 4b). Moreover, quantification of LC3bII/LC3bI and p62/Gapdh protein levels by Western blot analyses confirmed the effects of both AICAR and rapamycin treatments on these autophagic markers (Fig. 4c–e).

### AICAR and rapamycin affect skeletal muscle cells *in vitro*

**Figure 5:**
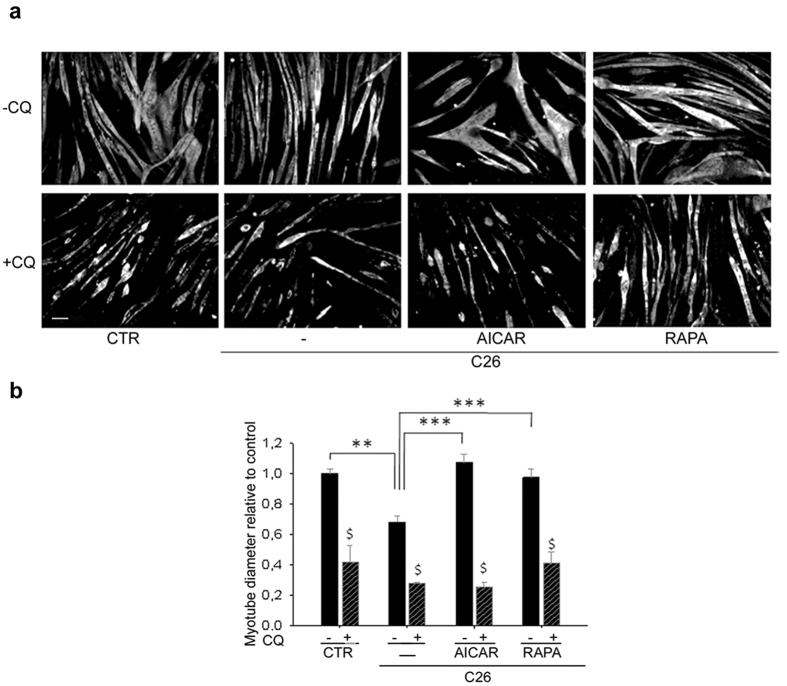
Both AICAR and rapamycin treatments counteract C2C12 myotube atrophy induced by C26 conditioned medium. (**a**) Representative immunostaining for myosin heavy chain on C2C12 myotubes cultured for 48 hours in the absence (CTR) or presence of C26-conditioned medium (C26), in the absence (—) or presence of 1 milliM AICAR, or 500 nanoM rapamycin, without (−CQ) or with (+CQ) 50 microM chloroquine. Scale bar = 20 microns. (**b**) Quantification of C2C12 myotube diameters in all the aforementioned conditions. n = 5. ^$^p < 0.0001vs -CQ; **p < 0.001; ***p < 0.0001.

Since both AICAR and rapamycin treatments were administered systemically, to prove that they directly affect skeletal muscle cells, we reproduced our experimental model *in vitro* by treating C2C12 myotubes with conditioned media derived from C26 cells. Forty-eight hours of treatment with C26-conditioned media induced approximately 30% of myotube atrophy *in vitro*, as quantified by measuring myotube diameter, whereas both AICAR and rapamycin treatments counteracted C26-induced muscle atrophy (Fig. 5). The effects of these treatments *in vitro* suggest that autophagy plays a role in controlling myotube size; to prove that autophagy is involved in the control of the myotube mass, we interfered with the autophagic flux by treating cells with chloroquine, an agent that prevents the fusion between autophagosomes and lysosomes^37^. A six-hour treatment with 50 microM chloroquine induced an accumulation of LC3b and p62 in myotubes (Supplementary Fig. S5), which is indicative of a block in the autophagic flux, and worsened myotube atrophy in all the conditions analyzed (Fig. 5).

## Discussion

Cachexia most commonly presents in lung- or gastrointestinal tract-cancer patients^38^, who can lose as much as 30% of their initial body weight. Since loss of strength and muscle mass are, together with involuntary loss of body weight^39^, the main features associated with cachexia, physical activity has proved to be a good therapeutic strategy to fight cachexia, thanks to its effects on both strength and muscle mass^40^. The molecular mechanisms underlying the physical activity-mediated rescue of cachexia are poorly understood. We show that all the major diagnostic features of C26-induced cachexia, such as loss of body weight, skeletal muscle mass and fiber size, can be counteracted by voluntary wheel running in mice. Voluntary running also improves skeletal muscle function in C26-bearing mice by raising the fatigue threshold, a relevant finding if we consider that fatigue is one of the most frequently reported complaints of cachectic patients^41^. It is noteworthy that C26-bearing mice did not develop anorexia and, thus, represent a pure cachexia model^42^. Conventional nutritional support cannot fully reverse cachexia, though a limited beneficial effect of appetite stimulants has been observed in mouse models^43^. We observed a significant increase in appetite in C26-bearing running mice associated with an improvement in muscle homeostasis, which suggests that exercise might have contributed to the improvement in muscle homeostasis by providing the aminoacids required for protein synthesis. Resistance exercise has traditionally been proposed to fight cachexia and other forms of muscle atrophy because it is a type of exercise that increases muscle protein content balance, mass, strength and resistance to fatigue. Here, we show that wheel running, which shares the features of endurance training^44^, effectively counteracts muscle wasting; as opposed to activating pro-hypertrophic signaling pathways^14^, this type of exercise counteracts pro-atrophic NF-κB-dependent signaling^45^. We observed a significant increase in life span in wheel running C26-bearing mice. Strikingly, life span directly correlates with the amount of voluntary running in C26-bearing mice. Exercise prolongs survival in both tumor-bearing mice and humans. However, since cancer patients, particularly the elderly, are often unable to perform voluntary exercise, any therapy based on voluntary running or other physical aerobic activities has certain limitations for cancer patients with progressive muscular atrophy. It is hence important i) to experimentally test alternative interventions, such as functional neuromodulation (i.e. electrical stimulation training of muscles) and ii) to identify the molecular pathways involved in voluntary running-mediated muscle rescue in cachexia. The latter is not merely due to an exercise-mediated shift toward a more oxidative fiber phenotype, as is shown by the fact that 19 days of wheel running did not significantly increase oxidative fibers^44^, which are considered to be more resistant to cancer cachexia^46^. Molecular analyses showed that wheel running reduces tumor-mediated atrogin 1 and MuRF1 gene expression, thus demonstrating that aerobic physical activity counteracts muscle atrophy by targeting the catabolic pathways of protein degradation. Pioneering analyses of the effect of a bout of wheel running (more than 5 km during the first night) demonstrated that muscle damage/regeneration/repair are accompanied by structural and molecular events associated with muscle apoptosis^47^,^48^. Apoptosis and inflammation share underlying healing (short-term) and/or damaging (long-term) processes: exercise may induce muscle adaptation to damage, but exposing severely cachectic tumor-bearing mice to exercise protocols might be deleterious. Consequently, pharmacological alternatives to physical activity should be considered.

Within this context, the results of our study show that one of the molecular pathways involved in cancer cachexia, i.e. autophagy, is directly responsible for the induction of skeletal muscle atrophy. It may thus be possible to use pharmacological treatments designed to trigger this cellular signal to improve muscle mass maintenance in cancer patients. We observed similar patterns of LC3bII and p62 protein levels in skeletal muscle biopsies from both colon carcinoma patients and C26-bearing mice, which were characterized by an accumulation of the lipidated form LC3b and the p62 protein. The fact that autophagocytosis normally degrades p62, whose levels thus become negligible, suggests that the autophagic flux is altered in the skeletal muscle of both animal models and human beings. In keeping with our findings, a recent study proved that autophagy is over-induced in skeletal muscle and contributes to muscle atrophy in cancer cachexia^32^. In that study, however, colchicine treatment, which blocks the autophagic flux after autophagosome formation^49^, anticipated the death of C26-bearing mice, thereby suggesting that a complete block in the autophagic flux is deleterious in cancer cachexia. The results of our study confirm the activation of the autophagic pathway, demonstrated by increased levels of the lipidated form of LC3b, and a delay in autophagosome clearance, demonstrated by the accumulation of p62, in the skeletal muscle of C26-bearing mice. Our *in vitro* observations with chloroquine also confirmed that a block in the autophagic flux is detrimental to skeletal muscle mass. The novelty of our findings stems from the fact that drugs promoting, and not inhibiting, the autophagic flux are effective against cancer cachexia. Despite being counter-intuitive, since autophagy may appear to be an additional catabolic pathway leading to sarcomere dismantling in cachexia, the model we propose suggests that it is of paramount importance to maintain balanced and efficient autophagy, which is dysregulated in cachexia, to improve muscle homeostasis. Autophagy is required at physiological levels for the removal of misfolded proteins and damaged organelles, and to prevent the accumulation of protein aggregates^50^. Autophagy has also been reported to play a key role in skeletal muscle homeostasis^27^. Skeletal muscles of autophagy-deficient mice progressively develop myopathy with age, and upon autophagy-triggering stresses, such as denervation or fasting, they severely degenerate owing to the accumulation of ubiquitinated proteins and damaged organelles^51^. Many studies have shown that an incorrect autophagic flux correlates with, and sometimes contributes to, skeletal muscle diseases such as Bethlem myopathy^52^, various muscular dystrophies^53^,^54^ and cancer cachexia^32^. The fact that treatment with two different drugs known to trigger autophagy, i.e. AICAR and rapamycin, improved muscle homeostasis in cancer cachexia strongly suggests that cachexia can be counteracted by triggering autophagy. AICAR has already been used to recover the autophagic flux in skeletal muscle and to counteract Duchenne muscular dystrophy in mice^55^. Rapamycin has instead been used as an immunosuppressant and anti-cancer drug^46^, though its effect on skeletal muscle wasting has never been investigated. Since AICAR and rapamycin, as well as other drugs that target AMPK such as metformin^56^, are currently being tested in cancer clinical trials^57^, these pharmacological treatments may be readily available for the translational applications deriving from our study. To prove that skeletal muscle cells are directly affected by AICAR, rapamycin and factors derived from C26 tumor cells, we reproduced cancer-derived muscle atrophy *in vitro* by treating myotubes with C26-conditioned media in the absence or presence of pharmacological treatments. Treatment with AICAR or rapamycin prevented C26-induced C2C12 myotube atrophy, thus proving that skeletal muscle cells are targeted by these drugs. Moreover, a block in the autophagic flux prevented the rescue of C26-induced myotube atrophy mediated by AICAR or rapamycin, thus indicating that both drugs regulate muscle homeostasis by promoting the autophagic flux.

**Figure 6:**
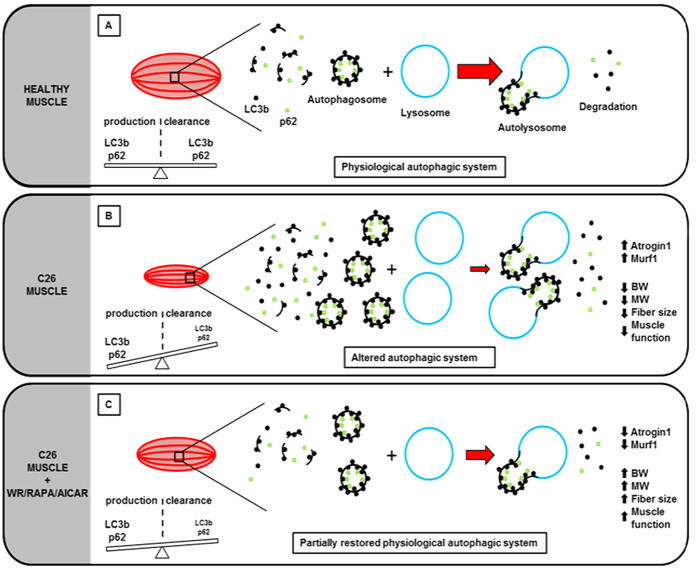
Exercise, AICAR and rapamycin counteract muscle wasting in cancer cachexia and modulate an adequate autophagic flux level. (**A**) In physiological conditions, a balance between autophagosome production and clearance maintains an adequate autophagic flux (red arrow) in the muscles, mirrored by basal levels of LC3bII (black dots) and p62 (green dots) expression. In cachectic muscles from C26-bearing mice (**B**), a strong accumulation of both LC3bII and p62 proteins points to an unbalanced autophagosome production/clearance ratio. This correlates with the pathophysiological features observed in cancer-related muscle wasting, including overexpression of Atrogin1 and Murf1 genes, as well as a decline in body weight (BD), muscle weight (MW), fiber size and muscle function. Spontaneous wheel running, or treatment with AICAR or rapamycin (**C**) counteracts cancer-related muscle wasting in tumor-bearing mice and induces a decrease in both LC3bII and p62 accumulation. Atrogin1 and Murf1 gene expression was restored to the basal levels observed in healthy muscles. This is associated with increased body and muscle weight and improved muscle function.

In conclusion, the pharmacological treatments used in our study and exercise share the ability to counteract cachexia and restore autophagic levels to those found in control healthy muscles. *In vitro* studies show that muscle cells are direct targets of both tumor-derived factors and exercise mimetics involved in the rescue of muscle mass homeostasis through a process involving the autophagic flux. A graphical abstract of our findings is shown in Fig. 6. To date, we cannot establish a causative role between the accumulation of autophagic markers and worsened cachexia, nor can we rule out the possibility that exercise and pharmacological treatments ameliorate C26-induced cachexia through indirect effects on muscle. In this regard, voluntary running was recently demonstrated to directly counteract tumor growth in tumor-bearing mice by modulating the inflammation of the tumor microenvironment^58^ However, our study provides unequivocal evidence showing that physical activity or exercise mimetics help to preserve muscle mass in the presence of a tumor and should be taken into consideration when planning therapies to treat cancer cachexia.

## Methods

### Mice and patients

Cachexia was induced by subcutaneous grafting of a 0.5 mm^3^ fragment of colon carcinoma (C26, obtained from the National Cancer Institute) in the dorsal region of 7-week-old BALB/c female mice (Charles River, Wilmington, MA), as previously described^29^. Unless otherwise specified, animals were sacrificed 19 days following tumor transplantation, on the basis of our evidence from survival curves showing that the vast majority of animals are still alive at this time point, even in the presence of overt cachexia. Mice were treated strictly according to the guidelines of the Institutional Animal Care and Use Committee, and to relevant national and European legislation, throughout the experiments. All the experimental protocols were approved by the Organ for Prevention and Wellbeing of Animals (OPBA) of the University of Palermo and by the Charles Darwin ethics committee n°5 in Paris and sent to the respective Ministries of Research in the two countries. Informed consent was obtained from patients for muscle biopsies.

### Exercise protocols and environmental enrichment

Wheels for rodents, with a diameter of 15 cm, were purchased in general customer pet shops. DC-9 tachometers were purchased from Decathlon. The mice were kept individually in cages that were identical, the only exception being that those of the WR mice were equipped with a wheel, as described in^44^, and were allowed to exercise ad libitum from the first day of the experiment, i.e. from the day of transplantation in tumor-bearing mice so as to mimic clinical studies in which physical activity started at the time of diagnosis. Since wheel-running activity may represent an environmental enrichment^30^, we set up additional, custom-made cages, characterized by the presence of novel objects, separate food reserves and tunnels, designed in such a way as to represent an environment enrichment^59^, to which a wheel was or was not added.

### Drug administration

Mice were treated daily by means of an intraperitoneal (IP) injection of 250 mg/Kg AICAR water solution (9 mice) or of a vehicle (18 mice), or of 2 mg/kg rapamycin in physiologic solution containing 1% ethanol (12 mice), or of a physiologic solution containing 1% ethanol (8 mice, which were subsequently pooled with the other vehicle group since no statistically significant difference between these two groups was detected in any parameter throughout the study). Treatments with both AICAR and rapamycin started on the day after tumor transplantation and stopped at sacrifice. All the groups in a given experiment were sacrificed on the same day, which was selected depending on the extent of cachexia developed by C26-bearing, non-treated animals. The latter were considered cachectic when they had lost at least 10% of their body weight.

### Histological and immunofluorescence analyses

TA muscles were dissected, embedded in tissue-freezing medium (Leica, Wetzlar, Germany) and frozen in liquid nitrogen-cooled isopentane. Cryosections (8 μm) were obtained using a Leica cryostat. Transverse cryosections of TA muscles were fixed in 4% paraformaldehyde for 10 min at room temperature. For p62 immunofluorescence analyses, muscles were permeabilized with 0.2% Triton in PBS for 30 min. After incubation with 1% BSA (Sigma, St. Lous, MO) for 30 min, samples were incubated with a 1:100 dilution in 1% BSA of polyclonal rabbit anti-laminin antibody (Sigma) or a 1:50 dilution in 1% BSA of anti-p62 guinea pig antibody (Progen) overnight at 4 degrees, followed by incubation with a 1:500 dilution in BSA of anti-rabbit-Alexa 488 or anti-guinea pig-Alexa 488 (Life Technologies) secondary antibodies for 1 hour at room temperature. 0.5 ug/ml Hoechst 33342 (Sigma) was used to stain nuclei.

### Morphometric analyses

Cross-sectional areas of TA myofibers were measured by using ImageJ software, freely availabe at http://imagej.nih.gov/ij/docs/intro.html. The whole muscle cross-sections from seven AICAR-treated mice or from seven vehicle-treated mice, and from five rapamycin-treated mice or from seven vehicle-treated mice were analyzed.

Myotube atrophy was quantified by measuring myotube diameter using ImageJ software. For each replicate sample, ten photomicrographs, each containing approximately 100 myotubes, were analyzed and the median myotube diameter (measured at the level of the maximum diameter displayed by each myotube) was calculated.

### Functional analyses

Fatigue time was measured as previously described. Briefly, the EDL muscle was mounted vertically in an ASI 300 b Dual-Mode actuator/transducer. To induce isometric fatigue, muscles were subjected to a series of closer trains of pulses (0.4 s train of 120 Hertz pulses) and the time required to halve the value of their own maximum strength was calculated.

### RNA extraction and Real-time PCR analyses

Total RNA was isolated and purified from 30–50 mg of TA muscle using Trizol Reagent (Invitrogen), according to the manufacturer’s protocol. One microgram of total RNA was converted to cDNA using the QuantiTect Reverse Transcription Kit (Qiagen). Real-time PCR was performed with the SDS-ABI Prism 7500 (Applied Biosystem, Life Technologies, Monza, IT), using the Sybr Green reaction mix (Applied Biosystem).

The following primer sequences were used:

atrogin1 for: GCA AAC ACT GCC ACA TTC TCT C

atrogin1 rev: CTT GAG GGG AAA GTG AGA CG

MuRF-1 for: ACC TGC TGG TGG AAA ACA TC

MuRF-1 rev: CTT CGT GTT CCT TGC ACA TC

myostatin for : TGC TGT AAC CTT CCC AGG ACC A

myostatin rev: GTG AGG GGG TAG CGA CAG CAC

GAPDH for ACC CAG AAG ACT GTG GAT GG

GAPDH rev CAC ATT GGG GGT AGG AAC AC

Five control healthy mice, six C26-bearing mice, six C26 WR, six vehicles, and five mice treated with either AICAR or rapamycin were analyzed.

### Western blot analyses

TA muscles were dissected, minced and homogenized in 0.4 ml of lysis buffer (50 mM Tris-HCl pH 7.4, 1 mM EDTA, 150 mM NaCl, 1% TRITON) supplemented with protease and phosphate inhibitors (complete Mini EDTA free and PhosSTOP, Roche). Cryosections from frozen rectus abdominis muscle biopsies of colon carcinoma patients were lysed in the same buffer as described above and used for further analyses. Proteins (30–50 micrograms) were separated by means of SDS-PAGE with varying % of acrylamide, depending on the protein molecular weight to be analyzed, and transferred to PVDF membrane (Invitrogen). Unspecific bindings were blocked in 5% non-fat dry milk (Nestlé) in TBST 1× (20 mM Tris, 137 mM NaCl, 0.1% Tween 20) for 1 hour at room temperature; membranes were then incubated at 4 °C, overnight, with primary antibody diluted in 5% BSA (Sigma) in TBST. The following primary antibody and dilutions were used: P-mTOR S2448 (Cell Signalling) 1:100; mTOR (Cell Signalling) 1:100; P-AMPKalpha T172 (Cell Signalling) 1: 1000; AMPKalpha (Cell Signalling) 1:1000; p-Acetyl-CoA Carbossylase S79 (Cell Signalling) 1:100; LC3b (Cell Signalling) 1:1000; p62 (Sigma) 1:10000; GAPDH (Santa Cruz) 1:10000; tubulin (Sigma) 1:10000. After washing in TBST, membranes were incubated for 1 hour at room temperature with HRP-conjugated anti-mouse or anti-rabbit secondary antibodies (Biorad) diluted 1:10000 in TBST, and signals were detected using ECL reagent (Advasta). Intensity of Western blot signals was quantified by using ImageJ software. Five control healthy mice, four C26 (—), four C26 WR, four AICAR-treated and four rapamycin-treated mice were analyzed.

### Statistical analyses

For each set of data, the first step entailed verifying the normal distribution of the data and their homoscedasticity by means of Levene’s test. If the data were homoscedastic, one- or two-way analysis of variance (ANOVA) was used, followed by Tukey’s HSD as a post-hoc test. If data were not homoscedastic (and in case of an unsuccessful transformation attempt), the data were analyzed using non-parametric tests, such as the Kruskal-Wallis test. Student’s t-test was used when only two treatments were compared. A Cox model was used to treat and analyze the survival curves. The specific test used and the global effects are indicated in the Results, while the specific post-hoc test used and the significance level of specific comparisons are indicated in the Figure legends, which also indicate the number of samples analyzed. All values are expressed as mean ± standard error of the mean (SEM) or as median ± semi-interquartile range, as appropriate.Both ISSP and VassarStats, a statistical computation website that is available for free at http://vassarstats.net/, were used as softwares for the statistical analysis.

### *In vitro* experiments

C26-conditioned medium was obtained by culturing confluent C26 cells (CLS Cell Lines Service GmbH, Eppelheim, Germany) for 2 days in serum-free DMEM (this C26 cell-conditioned medium was diluted 1 to 5 (20% final concentration) to treat C2C12 cultures (DMEM stored for 2 days at 37 °C used for controls). C2C12 cells were seeded at 20000 cells/cm2 and cultured in growing conditions (GM, DMEM containing 1% glutamine, 2% HEPES, 0.5% gentamicin, supplemented with 10% FBS, SIgma); the following day the cells were shifted to DMEM supplemented with 2% horse serum (DM, Sigma) and induced to differentiate for 4 days. C2C12 myotubes were cultured for 2 additional days with DM, in the absence or presence of 20% of C26-conditioned medium, with or without 1 mM AICAR (Sellckchem) or 50 nM rapamycin (Sellckchem) in 0.0025 DMSO solution. To block the autophagic flux, 50microM cloroquine (SIGMA) was added for 6 hours at the beginning of the treatments described above to 4-day myotube cultures; the samples were then extensively washed and incubated with the corresponding treatment for the remaining time until the end of the experiment.

### Immunofluorescence analyses

Cells were fixed in 4% PFA for 10 min at RT, permeabilized with 1% BSA plus 0.2% Triton in phosphate-buffered saline (PBS) and blocked in 5% goat serum (SIGMA) in PBS for 1 hour. Cells were incubated with a 1:50 dilution of p62 (Progen), or 1:50 dilution of LC3b (Cell Signaling), or 1:10 dilution of MF20 (Hybridoma Bank) in 1% BSA in PBS overnight at 4 degrees. Primary antibody incubation was followed by incubation with a 1:500 dilution in 1% BSA of secondary antibody anti-guinea pig, anti-rabbit or anti-mouse Alexa (Life technologies) for 1 hour at room temperature. 0.5 μg/mL Hoechst 33342 (SIGMA) was used to stain nuclei.

## Additional Information

**How to cite this article**: Pigna, E. *et al*. Aerobic Exercise and Pharmacological Treatments Counteract Cachexia by Modulating Autophagy in Colon Cancer. *Sci. Rep*. **6**, 26991; doi: 10.1038/srep26991 (2016).

## Supplementary Material

Supplementary Information

## Figures and Tables

**Table 1:** Wheel-running activity.

	Total distance (Km)	Partial distance* (Km)	Distance/day* (Km/d)	Time* (h:min)	Speed* (Km/h)
CTR WR	145 ± 11	51 ± 4	10.4 ± 0.3	20:59 ± 4:30	2.1 ± 0.1
C26 WR	105 ± 20	31 ± 10	6.3 ± 1.9	13:29 ± 6:20	1.9 ± 0.1

Control mice (CTR) and C26-bearing mice (C26) were kept in wheel-equipped cages and performed 19 days of voluntary wheel-running (WR) activity, which was individually recorded. *parameter values are based on the last five days, which correspond to a plateau phase of exercise activity and to overt cachexia in C26-bearing mice. The means ± SEM are shown. Tumor-bearing mice perform voluntary wheel running even in advanced stages of the disease.

**Table 2:** Mouse body weight according to treatments.

	Initial body weight (g)	Final body weight (g)
CTR	18.3 ± 0.4	19.5 ± 0.5**
CTR WR	18.7 ± 0.6	19.8 ± 0.4*
C26	18.7 ± 0.4	15.6 ± 0.4**
C26 WR	18.4 ± 0.34	19.7 ± 0.4^##^
C26 AICAR	18.9 ± 0.6	18.5 ± 0.9^#^
C26 RAPA	19.4 ± 0.8	19.4 ± 0.6^###^

Mice were divided into the following six groups: control mice (CTR); wheel-running mice (WR); tumor-bearing mice (C26); tumor-bearing, running mice (C26 WR); tumor-bearing, AICAR-treated mice (C26 AICAR); tumor-bearing, rapamycin-treated mice (C26 RAPA). All the treatments lasted 19 days, as explained in detail in the Methods, and the initial and final body weight were measured (the final weight of C26-bearing mice was equal to the carcass weight, i.e. total body weight minus tumor weight). Two-way ANOVA for repeated measures revealed no effect of WR on the body weight of healthy mice (control) but a significant effect of the presence of the tumor on the body weight of C26-bearing mice. In addition, ANOVA showed that the treatments had a significant effect in rescuing body weight. Multiple comparisons by Tukey’s HSD tests were used as post-hoc tests: **p < 0.0005 vs initial; *p = 0.001 vs initial; ^##^p < 0.0005 vs C26 final; ^#^p = 0.001 vs C26 final. C26 WR is NS vs C26 AICAR or C26 RAPA. The means ± SEM are shown; 9 < n < 34 for each group. The results show that each treatment significantly counteracts the C26-induced body weight loss.
