# Supplementary material for: Aerobic Exercise and Pharmacological Treatments Counteract Cachexia by Modulating Autophagy in Colon Cancer
Source: Sci Rep. 2016 May 31;6:26991. doi: 10.1038/srep26991 (PMC4886631; doi:10.1038/srep26991)
Supplement: Supplementary Information [file srep26991-s1.pdf]

Aerobic Exercise and Pharmacological Treatments Counteract Cachexia by Modulating Autophagy in Colon Cancer

Eva Pigna[1], Emanuele Berardi[1,2], Paola Aulino[1], Emanuele Rizzuto[1], Sandra Zampieri[3,4], Ugo Carraro[3,4], Helmut Kern[4], Stefano Merigliano[5], Mario Gruppo[5], Mathias Mericskay[6], Zhenlin Li[6], Marco Rocchi[7], Rosario Barone[8], Filippo Macaluso[8] , Valentina Di Felice[8], Sergio Adamo[1]*, Dario Coletti[1,6] and Viviana Moresi[1]

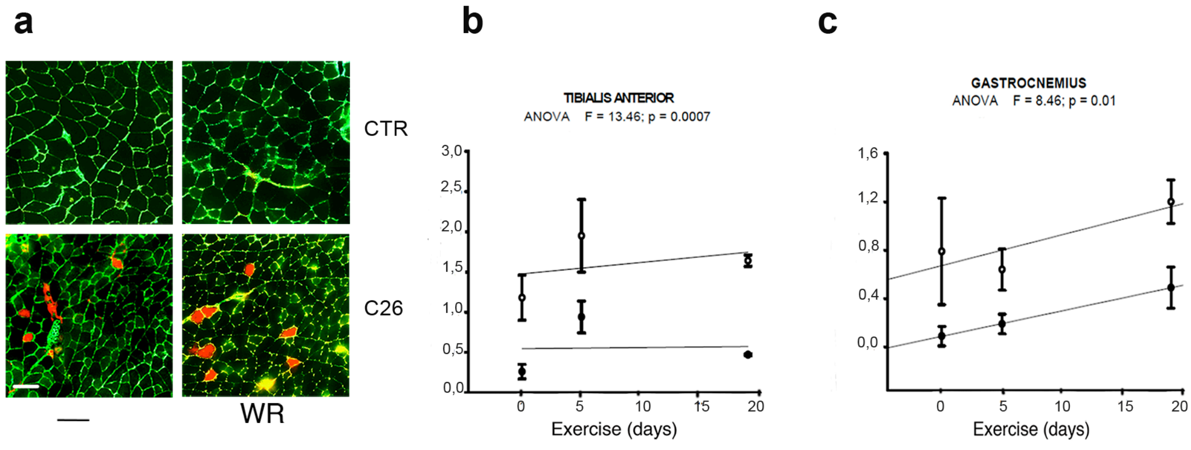

Supplementary Figure S1. C26 tumor but not wheel running induces muscle fiber damage.

(a) Representative immunostaining for laminin (green) on TA muscle in the absence of C26 tumor (CTR) or 19 days after C26 transplantation (C26), in the absence (-) or presence (WR) of wheel running, showing the presence of EBD^+ muscle fibers in red in C26-bearing mice. Scale bar = 20 microns. (b) Quantification of the EBD^+ muscle fibers in tibialis cross-sections from mice exposed to either 5 or 19 days of wheel running (exercise) in the absence (filled circles) or presence (open circles) of C26 (Two-way ANOVA $F = 13.46$; $p = 0.0007$). (c) Quantification of the EBD content normalized by DNA content in extracts obtained from the gastrocnemius, from mice exposed to either 5 or 19 days of wheel running (exercise) in the absence (filled circles) or presence (open circles) of C26 (Two-way ANOVA $F = 8.46$; $p = 0.01$).

a

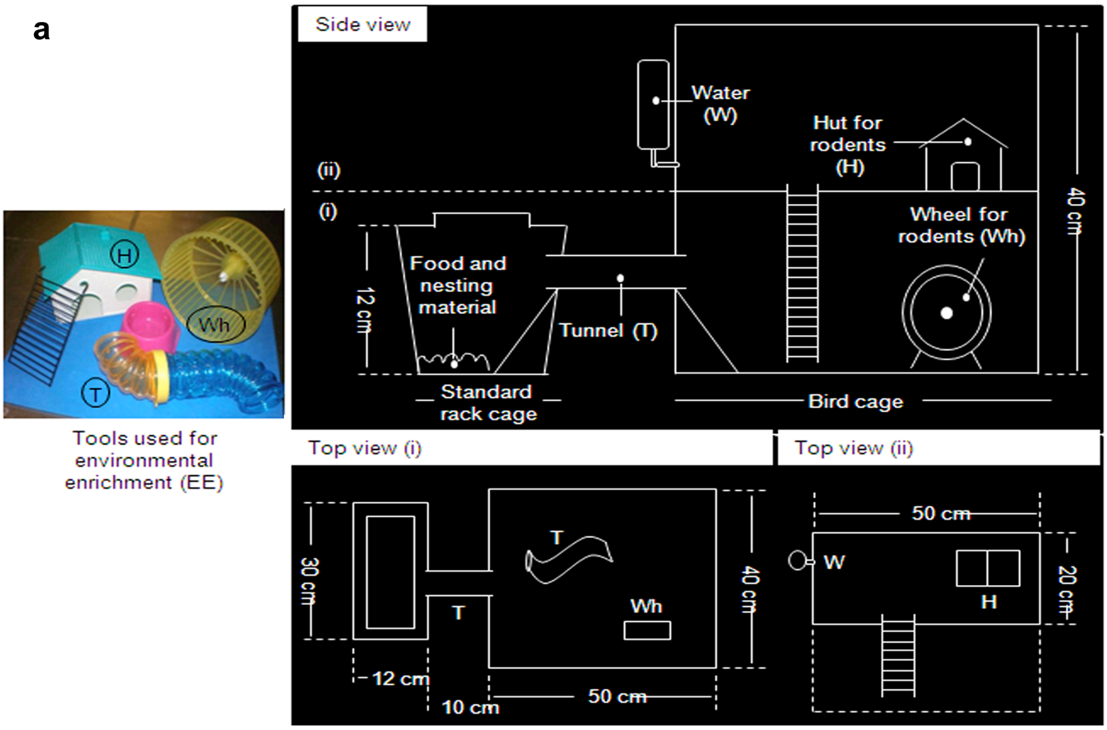

b

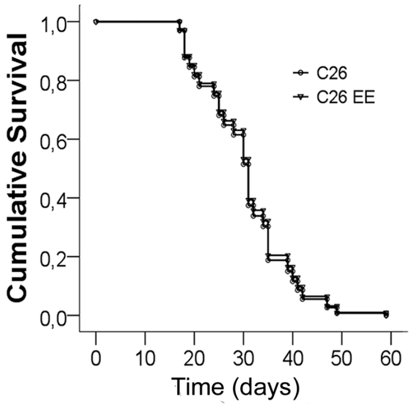

Supplementary Figure S2. Environmental enrichment system.

(a) Schematic drawing of the cages used for environmentally-enriched (EE) cages, either in the absence or presence of a wheel. (b) Survival curves derived from Cox model for statistical analysis of C26-bearing mice in the absence (C26, circles) or presence (C26 EE, triangles) of environmental enrichment. Environmental enrichment did not affect survival of C26-bearing mice. n = 9 for each group.

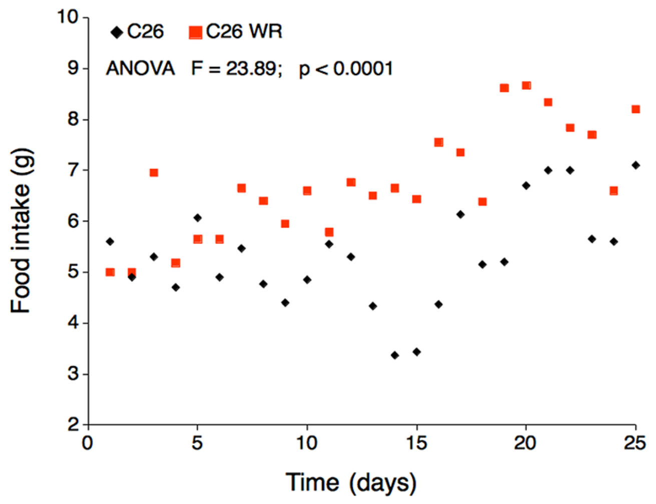

Supplementary Figure S3. Effects of wheel running on food intake kinetics by tumor-bearing mice.

Food intake kinetics of C26-bearing mice in the absence (C26) or presence (C26 WR) of wheel running. ANOVA shows a significant increase in food intake in running, tumor-bearing mice (C26 WR) compared with the matched population of non-running tumor bearing mice (C26) ($F = 23.86$; df 1; $p = 0.0001$).

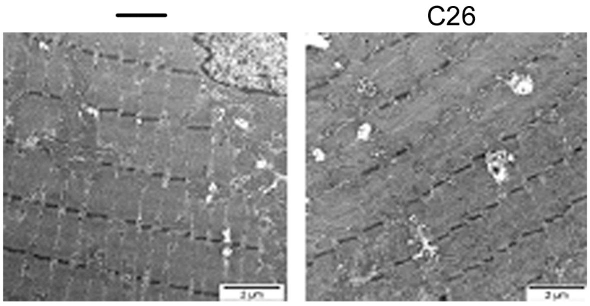

Supplementary Figure S4. C26-bearing mice exhibit accumulation of autophagosome-like structures.

Transmission electron microscopy images of skeletal muscle showing a representative myofiber from healthy (-) and C26-bearing (C26) mice. The latter displays classical double membrane autophagosomes with undigested cytosolic material. Scale bar = 2 microns.

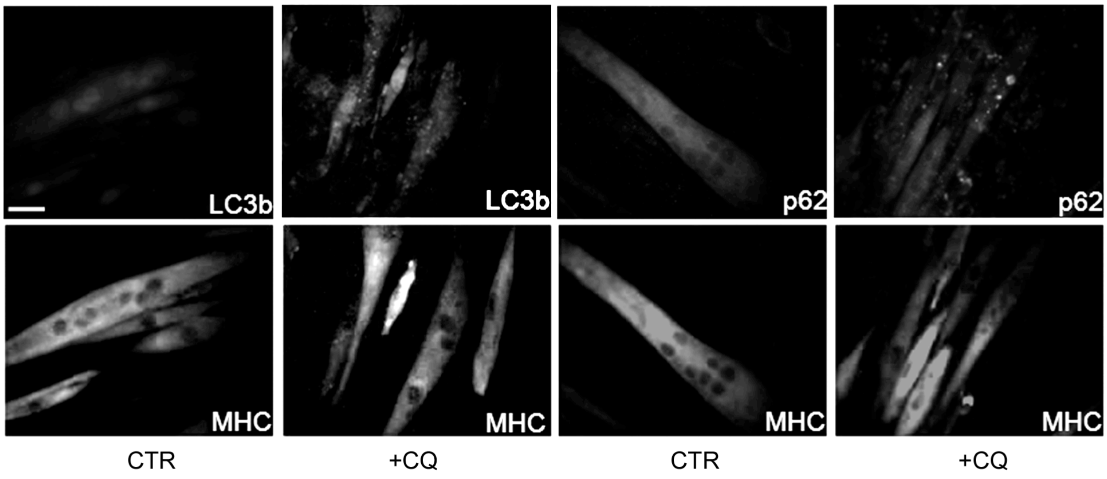

Supplementary Figure S5. Chloroquine treatment induces accumulation of autophagosomes in C2C12 myotubes.

Representative immunofluorescence analyses for LC3b and p62 in C2C12, myosin heavy chain (MHC) positive myotubes, in the absence (CTR) or presence of 6-hour 50 microM chloroquine (+CQ) treatment. Scale bar = 20 microns.

Supplementary Table 1. Clinical features of colon carcinoma patients included in the study

#	gender	age	BMI (Kg/m^2)	TOT PRT (g/L)	ALB (g/L)	TRANS-TYR (mg/L)	CK (U/L)	RCP (mg/L)	LEU $x10^9$	NP	CT	MP (%)	FF (%)
KC 14	M	59	NA	NA	48.0	285	75	1.6	NA	NA	NA	3	62
KC08	F	77	NA	NA	39.7	174	30	3.2	NA	NA	NA	1.7	49
KC04	F	28	NA	NA	41.5	165	57	1.9	NA	NA	NA	1.0	57
KC31	M	61	NA	NA	49.0	237	61	2.9	NA	NA	NA	4.7	NA
K0 53	M	67	26.0	71.3	38.2	156.2	50	5.2	7.49	N	N	15	33
K0 46	F	55	28.0	70.6	33.9	163.6	56	1.5	7.07	N	N	37	67
K0 40	M	59	24.2	74.1	42.8	149.8	34	3.0	3.88	N	N	16	76
K0 17	F	70	30.1	73.3	30.8	153.9	158	NA	8.82	N	N	4.5	43
K0 28	F	69	22.8	NA	38.5	189.4	36	1.6	NA	Y	Y	5.5	53
K0 21	F	69	26.6	70.1	32.5	148.9	103	3.5	6.36	N	N	5	65
K0 26	M	71	24.2	67.0	33.1	95.1	58	3.2	5.55	Y	Y	33	57
K0 14	M	60	24.6	NA	NA	NA	NA	1.02	5.22	Y	N	39	49

Healthy subjects (KC), used as controls, and colon carcinoma patients (K0) were randomly included in the study and muscle materials of bioptic origin used for the Western blot analyses. The order of the subjects listed in the table, from top to bottom, is the same as that loaded in the Western blot analyses, from left to right. Abbreviations: biopsy number (#); body mass index (BMI); serum parameters: total protein content (TOT PRT), albumin (ALB), transthyretin (TRANS-TYR), creatin kinase (CK), reactive C protein (RCP); leukocytes (LEU); neoplasia (NP); chemotherapy (CT); myopathy (MP: % of muscle fibers with centrally located nuclei); fast fiber (FF, % of muscle fibers expressing fast MHC).

Supplementary Table 2. Pathological classification of colon carcinoma tumours

		Pathological classification					
**#**	**Site of cancer**	**Grading**	**pT**	**pN**	**N+**	**N-**	**pM**
K053	sigma	G1	2	0	0	9	0
K046	colon	G3	3	0	0	26	0
K040	rectus	G2	2	0	0	23	0
K017	rectus	G2	3	2	12	4	0
K028	colon	G1	1	0	0	4	0
K021	colon	G2	3	1	2	9	0
K026	sigma	G3	4	0	0	15	0
K014	rectus	/	*in situ*	0	0	3	0

The Table shows the sites of neoplasia and the pathological classification of the surgically resected specimens. Grading describes the grade of differentiation between cancer cells and normal cells, ranging from 1 to 4, where 1 indicates normal cell-like appearance, while 4 indicates poor differentiation. Abbreviations: biopsy number (#); pathological tumor (pT), pathological lymph node (pN); lymph node (N); metastasis (M). pT describes local tissue infiltration of the primary tumor (mucosa, submucosa, muscularis, serosa); N describes the degree of spread of tumor cells to regional lymph nodes (0 absent); pM describes the presence of distant metastases. All the patients underwent surgery between 2008 and 2010.
